# MACAW: An Accessible Tool for Molecular Embedding and Inverse Molecular Design

**DOI:** 10.1021/acs.jcim.2c00229

**Published:** 2022-07-20

**Authors:** Vincent Blay, Tijana Radivojevic, Jonathan E. Allen, Corey M. Hudson, Hector Garcia Martin

**Affiliations:** † Biological Systems and Engineering Division, Lawrence Berkeley National Laboratory, Berkeley, California 94720, United States; ‡ Biofuels and Bioproducts Division, DOE Joint BioEnergy Institute, Emeryville, California 94608, United States; § DOE Agile BioFoundry, Emeryville, California 94608, United States; ∥ Global Security Computing Applications, Lawrence Livermore National Laboratory, Livermore, California 94550, United States; ⊥ Sandia National Laboratories, Livermore, California 94550, United States

## Abstract

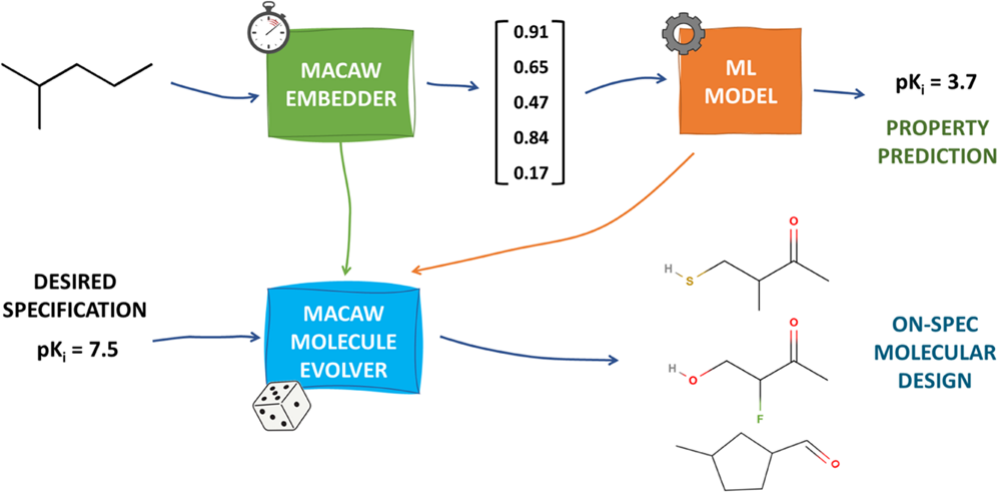

The growing capabilities of synthetic biology and organic chemistry demand tools to guide syntheses toward useful molecules. Here, we present Molecular AutoenCoding Auto-Workaround (MACAW), a tool that uses a novel approach to generate molecules predicted to meet a desired property specification (e.g., a binding affinity of 50 nM or an octane number of 90). MACAW describes molecules by embedding them into a smooth multidimensional numerical space, avoiding uninformative dimensions that previous methods often introduce. The coordinates in this embedding provide a natural choice of features for accurately predicting molecular properties, which we demonstrate with examples for cetane and octane numbers, flash points, and histamine H1 receptor binding affinity. The approach is computationally efficient and well-suited to the small- and medium-size datasets commonly used in biosciences. We showcase the utility of MACAW for virtual screening by identifying molecules with high predicted binding affinity to the histamine H1 receptor and limited affinity to the muscarinic M2 receptor, which are targets of medicinal relevance. Combining these predictive capabilities with a novel generative algorithm for molecules allows us to recommend molecules with a desired property value (i.e., inverse molecular design). We demonstrate this capability by recommending molecules with predicted octane numbers of 40, 80, and 120, which is an important characteristic of biofuels. Thus, MACAW augments classical retrosynthesis tools by providing recommendations for molecules on specification.

## 1 Introduction

Synthetic biologists and organic chemists are continuously expanding the universe of synthesizable small molecules. A few of these molecules could enable new pharmaceuticals, fuels, cosmetics, phytochemicals, pesticides, flavors and fragrances, or polymer precursors, if they have properties suitable to the application.^1,2^ For example, a new pharmaceutical molecule may be required to exhibit high binding affinity and specificity for its target receptor, adequate pharmacokinetic properties, and minimal toxicity. A new biofuel additive may be required to exhibit high octane number, a high flash point, and a low sooting index. Identifying molecules useful for an application amongst the myriad that could be synthesized is a lengthy and costly process. Advances in cheminformatic tools can help accelerate this process.

The data-driven prediction of molecular properties relies on a numerical description of molecules as input to machine-learning models.^3^ Traditionally, conventional molecular descriptors have been used to describe molecules.^4−7^ However, considerable time is often needed to select descriptors that are useful for the model at hand, amongst the thousands of descriptors available. Recently, deep learning methods have allowed the embedding or mapping of molecules into a numerical space (latent space) that can be used in modeling.^8,9^ However, these methods require large sets of molecules (typically >10^5^) to train deep networks^10^ and often need to be fine-tuned to the relevant chemical subspace using transfer learning, an artful process that requires time and expertise.^9,11^ More recently, molecular generative approaches have been developed, which are not constrained to predefined lists of molecules.^8^ However, the expertise required to use some of these tools and to direct the generation toward promising regions of the chemical space can limit their accessibility.

**Figure 1:**
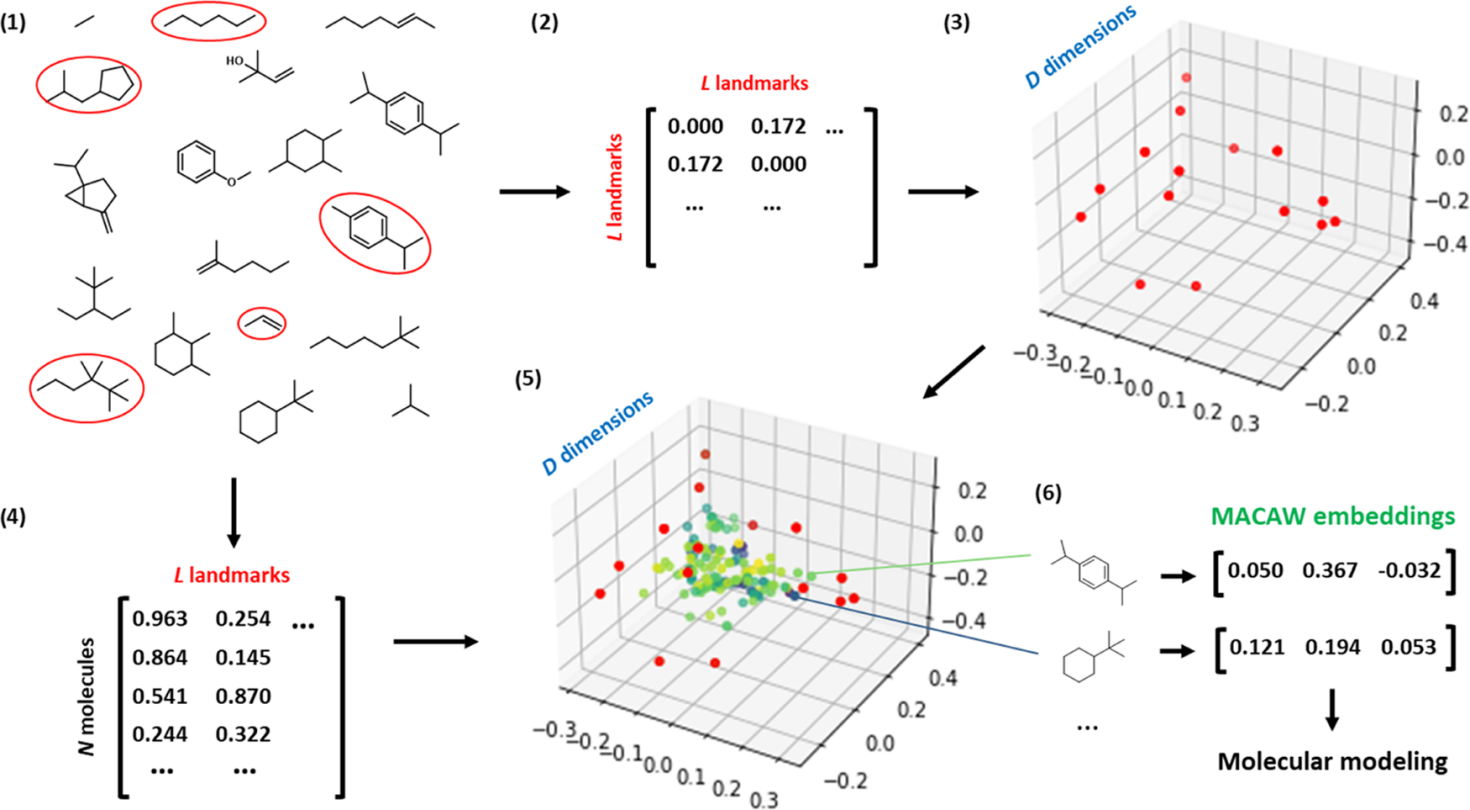
MACAW provides a simple and advantageous molecular embedding approach. (1) A small subset of *L* training molecules is defined as landmark molecules (circled in red). (2) Distances are computed between every pair of landmark molecules through any of the many distance metrics available for this end. (3) A projection of the landmark molecules onto the desired number of dimensions, *D*, is computed, which tries to preserve the relative distances between the landmark molecules in the embedding space. (4) The distances between the rest of the molecules and the landmark molecules are computed. (5) The molecules are rapidly projected to the embedding space by triangulation. (6) Each input molecule is thus assigned a *D*-dimensional numeric vector or embedding. Each embedding coordinate can be regarded as a feature and can then be used for modeling tasks just like conventional molecular descriptors. New query molecules can be projected onto an existing MACAW embedding starting from step 4. If desired, the embedding space can also be leveraged for generative tasks (see Section 3.2).

**Figure 2:**
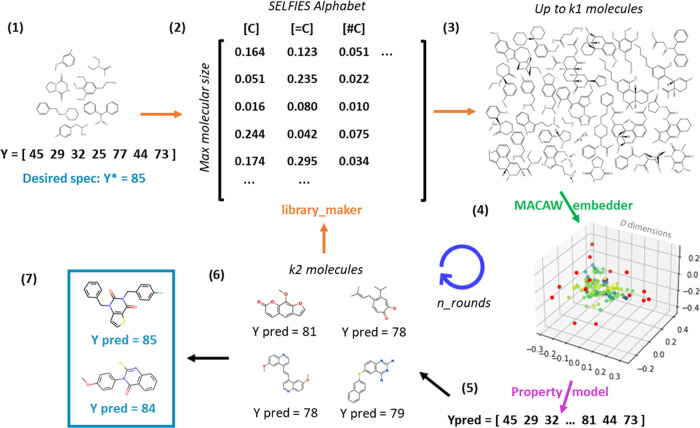
MACAW provides a novel way for inverse molecular design. (1) A dataset of molecules along with their property values and design specification are input to the library_maker function. (2) The library_maker function transforms SMILES into SELFIES and computes a probability matrix. The probability matrix illustrated is the relative frequency of each SELFIES symbol in each position of the molecular string, to which some normal noise is added. (3) Up to *k*1 molecules are generated using a probability matrix to choose a SELFIES symbol for each position and then converting the resulting string into SMILES for final output. (4) The molecules are then embedded using a given MACAW embedder (Figure 1), and (5) the embeddings are used as features to predict property values. (6) After considering the predictions in the current round and the *k*2 predictions from the previous round (if any), the *k*2 molecules closest to the specification are selected and used as input in another round of molecule generation. (7) After a number of rounds, the molecules closest to the design specification are returned. This algorithm is implemented in the library_evolver function, with the use of examples given in the Jupyter Notebook 4. This approach relies on the robustness of the SELFIES molecular representation, which allows concatenating random combinations of a set of SELFIES symbols and decoding them into SMILES strings with ∼100% validity.

In this work, we present Molecular AutoenCoding Auto-Workaround (MACAW), a cheminformatic tool to recommend molecules that fit a desired specification in a computationally efficient manner (inverse molecular design). MACAW combines two approaches to achieve this: a novel mapping of molecules onto a continuous vectorial space of selectable dimensions (embedding, Figure 1) and a novel way to generate and evolve molecules on a SELFIES alphabet (Figure 2).^12^ MACAW embeddings are richer in structural information and capture relevant molecular information more consistently than conventional molecular descriptors. We find that these characteristics enhance the predictive ability of machine-learning methods to predict molecular properties. The MACAW generative approach, based on these embeddings, can provide molecules with prespecified properties without the need for the expertise and time needed to train neural networks.

The application of MACAW is demonstrated for property prediction, virtual screening, and inverse molecular design. MACAW embeddings are used for the prediction of complex properties, including research octane number (RON), cetane number (CN), melting point (MP), and flash point (FP) of molecules, as well as the binding of small molecules to the histamine H1 receptor and the muscarinic M2 receptor. These properties are demonstrative of both biofuel and medicinal applications. We also demonstrate the use of MACAW embeddings in virtual screening: the rapid embedding and prediction of properties for tens of thousands of molecules enables the identification of molecules that bind strongly to the histamine H1 receptor but weakly to the muscarinic M2 receptor. Furthermore, MACAW embeddings can help recommend molecules with prespecified properties (inverse design problem). For this, we propose a directed evolution strategy in silico that involves a novel probabilistic molecular generator. This inverse design method avoids the need for the expertise and time to train a complex decoder network and is well-suited to the dynamic nature of MACAW embeddings.

Overall, MACAW enables successful molecular property modeling and inverse molecular design in a few lines of user code, at low computational cost, without the need for variable cleaning or selection, and with the flexibility of a dynamic molecular representation method.

## 2 Materials and Methods

### 2.1 Encoding

MACAW encodes molecules into numeric vectors that can be used as inputs to mathematical and machine-learning models. Thus, each molecule is mapped to a point in a *D*-dimensional space, in a way that molecules that are more similar to each other are located closer in the numerical space and molecules that are more dissimilar are located further away. MACAW offers different options to measure molecular similarity, which can be tuned to the problem at hand. An overview of the encoding pipeline is illustrated in Figure 1.

The encoding of molecules is done through the MACAW class. Initializing a MACAW object requires providing a list of molecules in SMILES format as input. The user can specify the number of *L* landmark molecules to use (optional *n*_landmarks argument, defaults to 50) and the desired dimensionality *D* of the embedding (optional *n*_components argument, defaults to 15), with *D ≤ L.* A subset of *L* molecules is then designated as landmarks (Figure 1.1). Landmark molecules are chosen at random (default) or, if property values are also provided (optional *Y* argument), they are picked after binning the molecules by property value (10 bins by default). Thus, a landmark molecule has the same probability of being chosen from each bin, regardless of the number of molecules in each. This binned landmark selection method is particularly recommended for skewed datasets. Other options include the choice of landmarks to be the molecules with the highest or lowest property values (optional Yset argument).

Distances between molecules in MACAW depend on a combination of molecular fingerprint type (or types) and a similarity metric. Molecular fingerprints describe molecules through a characteristic bit or Boolean vector representation. A wide variety of molecular fingerprints have been developed,^13^ involving, for example, assessing the presence or absence of patterns in the molecule, counting the number of certain motifs, or hashing or folding an intermediate vector into a fingerprint of the desired length, among others. On the other hand, similarity metrics capture in a single number the resemblance of a pair of bit vectors. A well-known example of these similarity metrics is Tanimoto similarity,^14^ which assesses the fraction of “on” bits that are common between two bit vectors, but there are many more.^15,16^

MACAW features a variety of distance metrics between molecules, enabled by rdkit. This is done by specifying a combination of molecular fingerprint type {morgan2, morgan3, rdk5, rdk7, featmorgan2, featmorgan3, maccs, avalon, atompairs, torsion, pattern, secfp, layered, daylight} and similarity metric {Tanimoto, dice, cosine, Sokal, Kulczynski, Mcconnaughey, Braun-Blanquet, Blay-Roger, Rogot-Goldberg, asymmetric, Manhattan}.^13^ MACAW also allows concatenating different types of fingerprints prior to their projection, which might strengthen the performance of the resulting embedding in some cases. For example, one can specify “pattern + atompairs” as the fingerprint type to achieve an embedding based on these two types of fingerprints. See the accompanying Jupyter Notebook 1 for additional examples. The similarity *s* between two molecules *m*_*i*_ and *m*_*j*_ is a scalar in the interval *s*(*m*_*i*_*,m*_*j*_) ∈ [0,1], which is then converted to a distance metric *d*(*m*_*i*_,*m*_*j*_) in MACAW using eq 1.
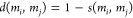
1Given the relative distances between pairs of molecules, they can be projected as points in a numerical space, while preserving these relative distances as much as possible. To preserve relative distances while reducing the number of dimensions, a multidimensional scaling (MDS) algorithm can be used.^17^ However, conventional MDS is computationally slow for large numbers of molecules. Instead, MACAW employs a landmark-MDS projection algorithm,^18^ which only requires computing the distances to a subset of landmark molecules (Figure 1.2,4). In this case, the landmark molecules are projected first using the classic MDS algorithm (Figure 1.3), while the rest of the molecules are projected afterward by triangulation (Figure 1.5). Alternative algorithms to landmark-MDS are also available in MACAW, including isomap projection (algorithm = “isomap”), principal component analysis (PCA) projection (algorithm = “PCA”), independent component analysis (algorithm = “ICA”), and factor analysis (algorithm = “FA”). Compared to MDS, the other projection algorithms give different weights to the distances to different landmarks; for example, in isomap projection, the algorithm tries to preserve the geodesic distances to neighboring landmarks only. In all cases, the embedder is computed based on the distances between landmark molecules. Then, it can be applied to any new molecules rapidly, with the embedding time scaling linearly with the number of molecules *N*, that is, . As a result, each molecule is mapped to a point in the MACAW embedding space, whose coordinates can be used as molecular features.

A MACAW instance can be used to embed any input molecule provided in SMILES format. In particular, when declaring a MACAW Python object, it has to be initialized through its train method. This method chooses and embeds the landmark molecules from the list of molecules supplied (steps 1, 2, and 3 in Figure 1). The MACAW instance can then be used to embed any list of *N* molecules (smiles) by applying the transform (smiles) method (step 4 in Figure 1). One can also initialize the embedder and embed the input molecules at once using the fit_transform (smiles) method. This method also avoids recomputing the pairwise distances between landmark molecules. The output of the transform or fit_transform method is an array of size *N* × *D*, with each row being the embedding of the corresponding molecule in the input list (see Jupyter Notebook 1). These embeddings can be used as predictors in machine-learning models, like those in scikit-learn,^19^ TPOT,^20^ or ART.^21^

MACAW has several hyperparameters that can be tuned to optimize the performance of the embedding. To simplify this choice, the function MACAW_optimus automatically explores a variety of fingerprint type and distance metric combinations and returns a recommended embedding for the problem at hand. This automated, heuristic selection is based on the performance of different embeddings in the cross-validation of a support vector machine (SVM) model. This functionality is illustrated in the accompanying Jupyter Notebook 1. The MACAW class also has several setter methods (set_type_fp, set_metric, set_*n*_components, and set_algorithm) that allow changing hyperparameters of the MACAW embedding while minimizing the computations needed. For example, changing the desired dimensionality of the embedding does not require recomputing distances. These methods can be useful to explore a variety of hyperparameters using a grid search, for example.

### 2.2 Datasets

#### 2.2.1 Biofuel Properties

A dataset of 194 molecules along with experimentally measured research octane number (RON) values was compiled from a variety of sources.^22−26^ A dataset of 545 molecules and their corresponding experimental cetane number (CN) values was compiled from the literature.^22,27^ We also modeled molecular melting points (28,266 molecules),^28^ yield sooting indices (610 molecules),^29^ and flash points (631 molecules).^27^

#### 2.2.2 Histamine and Muscarinic Receptor Binding

A set of 1214 compounds was evaluated for their binding affinity to the histamine H1 receptor using compounds retrieved from ChEMBL version 27 with binding assays used to measure binding *K*_*i*_ values. Similarly, a set of 1145 compounds from ChEMBL were evaluated against the muscarinic M2 receptor to measure binding *K*_*i*_ values. Assay data was collected from multiple publications, example studies include refs (30) and (31). The M2 receptor is considered in this work as an undesired off-target in the design of new H1 inhibitors.^32^

To discover new potential ligands specific against H1, we compiled a virtual library by combining the Enamine Antiviral Library (3200 compounds), the Enamine Discovery Diversity Set 10 (10,240 compounds), the Enamine Nucleoside Mimetics Library (290 compounds), and the Enamine Phenotypic Screening Library (5760 compounds), resulting in 19,490 screening compounds.

### 2.3 Conventional Molecular Descriptors

MACAW was compared to conventional molecular descriptors as an established method to numerically encode molecules. A set of molecular descriptors was computed with the free software rdkit 2020.09.4, using the custom function indicated in Jupyter Notebook 5. A larger set of molecular descriptors was computed using the commercial software alvaDesc 2.0.2. Descriptors that were invalid for any molecule were dropped, resulting in 196 rdkit descriptors and 2950 alvaDesc descriptors.

The informativeness of conventional molecular descriptors and MACAW embeddings were evaluated using mutual information (MI). The MI between a predictor and a property quantifies how informative the predictor is of the property of interest. MI can be regarded as a more general measure of association than Pearson correlation, as it also takes into account nonlinear dependence, which some machine-learning models are able to learn. More specifically, MI quantifies the reduction in information entropy of the target variable *Y* that is attained by knowing the predictor *X* (eq 2)
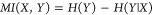
2where *H*(*Y*) is the marginal information entropy and *H*(*Y*|*X*) is the conditional information entropy.^33,34^ A value of zero indicates that *Y* and *X* are uninformative of each other, and a high value indicates that they are closely interrelated. For each molecular property dataset, a subset of 194 molecules and property values were sampled to estimate the MI using the mutual_info_regression function in scikit-learn 0.24.1.^19^

The performance of conventional molecular descriptors as regressors was evaluated (Figure S4 and Jupyter Notebook 5). Given the large numbers of conventional molecular descriptors that are typically computed (hundreds to thousands), a variable selection step was conducted to select a small subset of informative descriptors with which to build a model. The selection was carried out using a heuristic forward stepwise selection algorithm, which is commonly applied to conventional molecular descriptors.^35−37^ This algorithm tends to give good results, although it can be time-consuming, as it requires to train and evaluate *N* × *D* × *F* models, where *N* is the number of variables considered for the selection (several hundred in this case), *D* is the number of desired descriptors being eventually selected (15 in this case), and *F* is the number of cross-validation folds used to evaluate each model (5 in this case). Furthermore, the algorithm requires specifying a model for the intermediate variable selections. Since the optimal model is unknown a priori, a linear model was used for the variable selection to prevent overfitting. The 15 selected features were then used to train SVM models analogous to those trained on MACAW features, including the same hyperparameter optimization.

### 2.4 Property Modeling

We used support vector machines (SVMs) as the default model choice because the focus of this work is to show the general applicability of MACAW molecular embeddings to facilitate modeling and molecular design, rather than developing the best possible model for every property. SVMs offer a reasonable tradeoff between speed, flexibility, and ease of implementation, particularly for small and medium-size datasets. The models were built using scikit-learn 0.24.1.^19^ MACAW embeddings were computed and used as predictors. In all cases, we perform 10-fold cross-validation by splitting the dataset into validation and train sets, where the validation set is obtained by randomly splitting the whole dataset into 10 almost equal partitions and the train set is the remainder. For each of the folds, the model is trained on the train set and tested on the validation, such that the accuracy metrics, *R*^2^, mean absolute error (MAE), and root mean squared error (RMSE), are calculated for predictions on unseen data. Furthermore, tuning of the SVR models was done by grid search of hyperparameters (regularization parameter *C* and epsilon) considering the model’s 5-fold cross-validation performance on the training partition. Notably, the validation sets were not involved in the computation of the embeddings or the tuning of any hyperparameters for each of the folds. Property modeling examples are provided in the accompanying Jupyter Notebook 2.

### 2.5 Molecule Generation

We have developed an original method that generates libraries of molecules of arbitrary size in a probabilistic manner around an input set of molecules. This method is implemented in MACAW’s library_maker function (Figure S1). The method works by encoding the molecules input by the user into a one-hot SELFIES representation.^12^ A new molecule is generated by drawing SELFIES tokens from an alphabet in a probabilistic manner. The probabilities are specified by a matrix extracted from the input molecules (see below). The SELFIES tokens are concatenated to form a string, which is then decoded as a SMILES by the SELFIES interpreter. The process is repeated to generate as many molecules as desired; the resulting SMILES are then canonicalized and any duplicates are removed. The generated molecular library can be used as any other library and be embedded into the *D*-dimensional MACAW space.

To achieve a more robust production of valid molecules, by default, we use an alphabet of SELFIES tokens observed in the input molecules for which chemical valence rules are implemented in the SELFIES package (i.e., state-dependent derivation rules have been hard-coded). By default, molecules are generated with their SELFIES length drawn from a discrete distribution (by default, *p*(*n*) ∝ exp(*n*), where *n* is the length up to the maximum requested length max_len). If not specified, max_len will be set to the length of the longest SELFIES observed in the input.

The probability matrix is computed by counting frequencies of SELFIES tokens and then adding some noise. Different options on how the frequency matrix is constructed are available, which are specified by the algorithm input to library_maker:

- If algorithm = “position” (default setting), the matrix captures the frequency of each SELFIES token as a function of its position in the input SELFIES strings. The dimensions of the probability matrix will be (SELFIES alphabet length, max_len).
- If algorithm = “transition”, the matrix captures the frequency of each SELFIES token following another token. In this case, the probability matrix will be a square matrix of dimensions (SELFIES alphabet length, SELFIES alphabet length).
- If algorithm = “dual”, the matrix captures the frequency of observing a SELFIES token after another token in each specific position of the SELFIES string. In this case, the probability matrix will be a three-dimensional (3D) matrix of dimensions (SELFIES alphabet length, SELFIES alphabet length, max_len).

In all three cases, the resulting matrix of frequencies is normalized row-wise and is combined with a uniform probability matrix in an affine combination. The resemblance to the uniform probability matrix is controlled by the argument noise_factor. It can take values between 0 and 1, with higher values leading to a more random drawing of tokens (Figure S1).

The library_maker function is also able to generate molecules when no SMILES are provided as input. In this case, a predefined alphabet of tokens and a uniform probability matrix are used to generate molecules.

### 2.6 Hit Identification

To retrieve molecules satisfying a specification from an embedded library of molecules, two search algorithms (hit_finder and hit_finder_grad) have been developed that only require evaluating a few molecules in the library. The algorithms require providing a predictive model and a desired property specification along with the library of molecules. The algorithms avoid having to exhaustively evaluate the property model on the whole library, which may be useful for models that are expensive to evaluate (e.g., kernel-based models trained on a large dataset). The algorithms can be applied to the MACAW embedding of an existing molecular library or to a library generated with the library_maker function.

In the first search algorithm, hit_finder, the MACAW-embedded library is first organized for quick access using sklearn’s BallTree algorithm. This algorithm organizes a collection of points for lookup by partitioning the search space into a tree once, speeding up the retrieval of subsequent queries^19^ (Figure S2). The choice of the distance metric to build the tree is important as it affects which molecules are in the vicinity of any given molecule. For use with MACAW embeddings, we recommend the Manhattan distance (*p* = 1) over other Minkowski norms (*p* > 1), as this penalizes less molecules further away from the query seed molecule in some dimension, as long as they are relatively close in other dimensions. Taking this idea a step further, we introduce a custom *V*-distance, which is invoked when 0 < *p* < 1 and that is defined as follows using eq 3
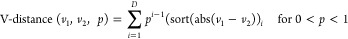
3where *v*_1_ and *v*_2_ are *D*-dimensional vectors, and the function sort arranges the elements of a vector in increasing order. Note that for *p* = 1, the *V*-distance equals the Manhattan distance metric.

Next, we take the *k*1 most promising molecules from the training dataset and consider their projection in the MACAW vectorial space. The most promising molecules in this context are those with property values closest to the desired specification provided by the user. Querying the BallTree, we then retrieve the *k*2 closest molecules to the *k*1 seed molecules (measured with the selected distance metric), retrieving a maximum of *k*1 × *k*2 library molecules. The property values of these molecules are predicted using the property model supplied, and the most promising molecular designs are returned to the user.

A second function, hit_finder_grad, is also provided, which leverages the smoothness of MACAW embeddings and the predictive model using a scipy gradient-based minimization algorithm. In this case, the algorithm is started *k*1 times from random molecules throughout the library to find points that minimize the square of the difference between the specification and the predicted property value. Next, the BallTree is queried for the *k*2 molecules nearest to each minimum and their property values are predicted. Finally, those molecules closest to the requested specification are returned to the user in SMILES format.

### 2.7 Inverse Design

We propose an original evolutionary strategy to recommend new molecules satisfying a desired design specification. The strategy involves an increasingly focused generation of molecules along with the selection of the most promising molecular designs. This process is achieved using MACAW’s library_evolver function (Figure 2), which requires as inputs a starting set of molecules in SMILES format, a MACAW embedder, a predictive property model, and the desired property specification. The use of this function is illustrated in the accompanying Jupyter Notebook 4.

The method encompasses a number of iterations or rounds (*n*_rounds = 8 by default). In each round, a set of molecules is used to seed the library_maker function described above. Up to *k*1 molecules are generated through library_maker (*k*1 = 3000 by default), which are then embedded using the MACAW embedder supplied, and their property values are predicted using the model supplied. The *k*2 molecules closer to the desired specification (*k*2 = 100 by default) are selected and carried over to the next round to seed a new library_maker run (these molecules are also included in the new dataset from which the most promising molecules will be selected). In total, up to (*k*1 × *n*_rounds) molecules are evaluated across the MACAW embedding space, sampled from regions of the space with predicted property values increasingly closer to the desired specification. The *n*_hits (10 by default) molecules closest to the desired specification in the final round are returned to the user along with their predicted property values.

## 3 Results and Discussion

### 3.1 Effect of Embedding Hyperparameters

The MACAW process of mapping discrete molecules onto a continuous numerical space involves three main steps (Figure 1 and Section 2). First, molecules are characterized in the form of a bit vector molecular fingerprint (e.g., rdk5, Morgan2, MACCS, Avalon, or Torsion). Second, these fingerprints are then used to compute relative distances between some of the molecules in the dataset, which is expected to extract the most relevant information from the fingerprints. Finally, a fast algorithm is used to place the molecules in the numerical embedding space in such a way that these relative distances are preserved as much as possible. The coordinates of the embedding can then be used as molecular predictors. In this process, there are some choices of hyperparameters that can be optionally specified.

**Figure 3:**
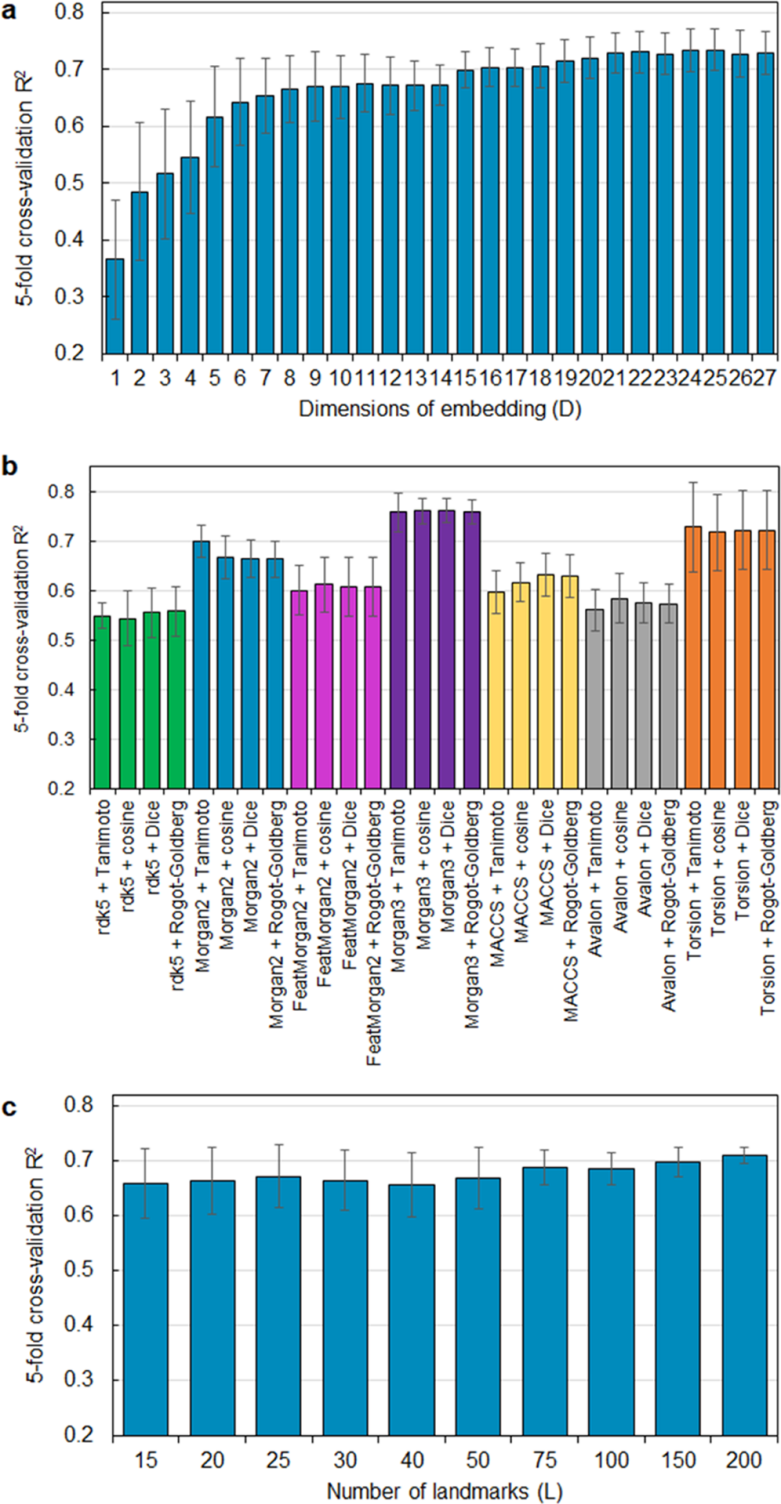
MACAW’s hyperparameters have limited impact on predictive capabilities, except for the choice of fingerprints and distance metric. Typical effect on the model performance of (a) the dimensionality of the MACAW embedding, (b) the fingerprint and distance metric used, and (c) the number of landmarks chosen. *D* = 10, *L* = 50, and Morgan2 fingerprints with Tanimoto similarity were used unless indicated otherwise. An SVR model with *C* = 100 and ε = 5 was used in all cases to model the cetane number dataset.

MACAW hyperparameters can be tuned to optimize the performance of the embedding for subsequent predictive modeling. The hyperparameters include the number of landmarks, the dimensionality of the embedding, and the choice of fingerprint type and similarity metric. Analyzing the effect of these hyperparameters (Figures 3 and S3), we found that using 15–25 MACAW embedding dimensions is sufficient for most problems. Using more dimensions for the MACAW embedding generally does not have a detrimental impact on model performance (Figures 3a and S3), at least when landmark molecules are chosen randomly as in the cases studied. On the other hand, the performance of the embeddings seems minimally affected by the choice and number of landmarks, as long as a sufficient number of them is used (Figures 3c and S3).

Different strategies to select landmarks have been proposed, with a random choice working well in many cases.^18,38^ In this case, robust performance can be obtained by choosing substantially more landmarks than required for the embedding (*L* ≫ *D*), as pointed out by the narrowing of the error bars in Figure 3c. However, when the distribution of property values in the dataset is skewed, like the yield sooting index dataset in this work, performance with random landmarks can suffer. With this in mind, we developed a new binned landmark choice strategy in MACAW (see Section 2). This landmark selection method often provides similar or better results than random choice and it is particularly recommended when the distribution of property values is skewed.

The choice of molecular distance can have a significant impact on the performance of the embedding (Figure 3b), and MACAW can automatically recommend a setting. The molecular distance is specified by a combination of fingerprint type and similarity metric, with the optimal choice depending on the problem. By default, MACAW uses Morgan fingerprints of radius 2 and a Tanimoto similarity metric. The setter methods in the MACAW class allow to efficiently vary the hyperparameters and conduct a grid search. Moreover, the function MACAW_optimus automates the selection of fingerprint and similarity metric for a given problem by assessing the performance of an SVM trained on different embeddings. Its use is illustrated in the accompanying Jupyter Notebook 1.

### 3.2 Efficient Prediction of Molecular Properties

**Figure 4:**
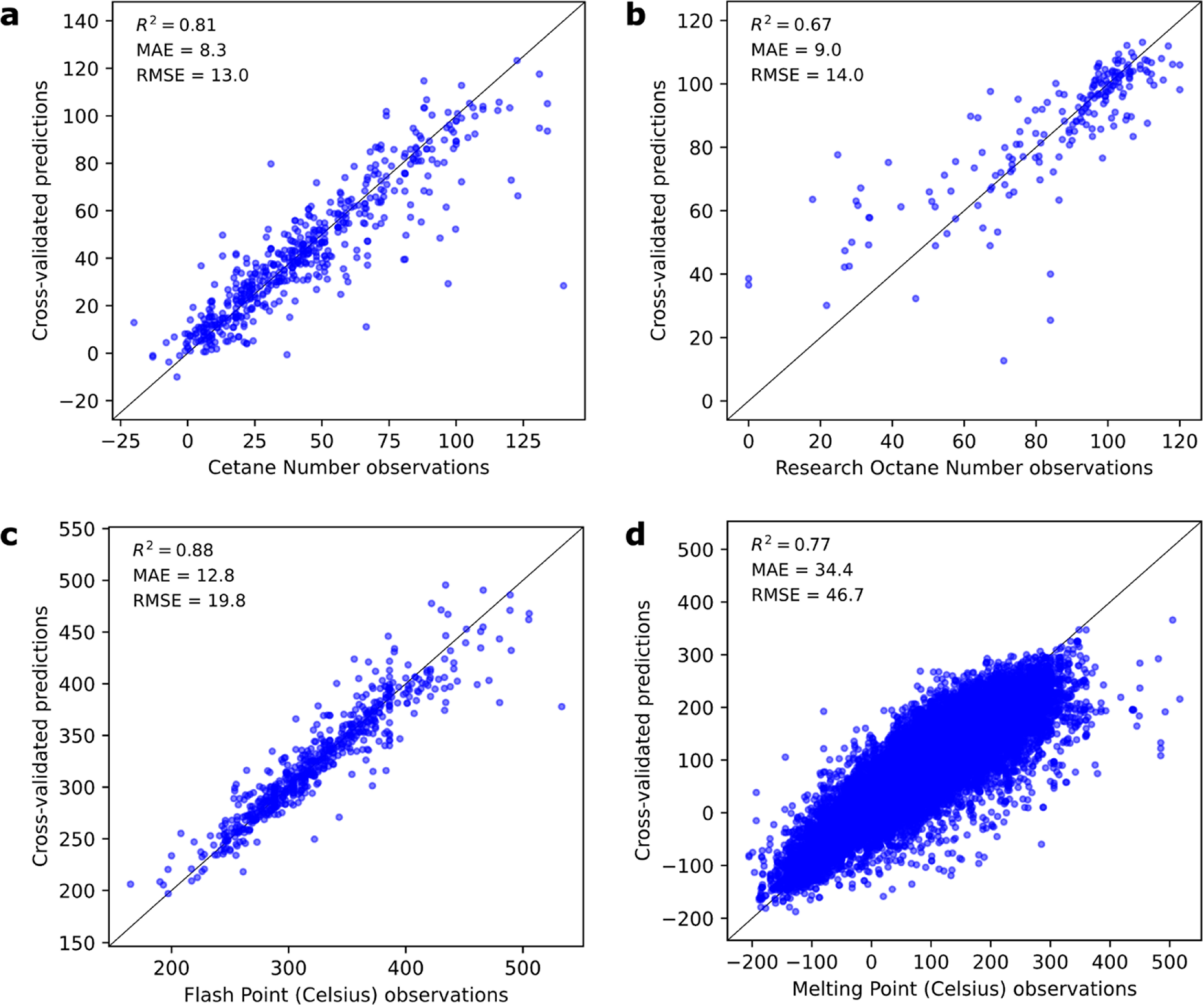
MACAW embeddings provide excellent molecular representations for predicting a variety of molecular properties. Observations and cross-validated predictions for different (bio)fuel properties are shown: (a) cetane number (AtomPairs + Rogot-Goldberg), (b) research octane number (AtomPairs + Dice), (c) flash point (Pattern + AtomPairs + Rogot-Goldberg), and (d) melting point (MACCS + Tanimoto). SVRs with radial basis function were trained in all cases. Predictive accuracy metrics are calculated using 10-fold cross-validation. See Jupyter Notebook 2 for details. The MACAW embeddings set the stage for predictions that are the same or better than conventional molecular descriptors (Figure S4).

Using MACAW embeddings, good performance could be easily obtained in a variety of property prediction tasks (Figure 4). For example, four different molecule datasets (cetane number, research octane number, flash point, and melting point) were embedded into 10-D or 15-D spaces using landmark molecules from the training datasets with the automated MACAW_optimus function. The features were used as inputs to train the support vector regressor (SVR). After grid search optimization of the regressor’s hyperparameters using nested cross-validation (see Section 2), *R*^2^ values on cross-validated predictions larger than 0.67 could be obtained for the four properties (Figure 4). These are very encouraging results despite the complex properties being modeled. Note that the flash point, cetane number, and octane number are all high-order properties of great interest for biofuels, for which accurate predictions from first principles are very challenging.^22,39^ These may be complex functions of other properties like vapor pressure, diffusivity, bond energies, thermodynamic properties, as well as the reactivity of the dozens of radical species and intermediates that may be generated from any given compound in the fuel combustion process.^40^ A comparison of MACAW embeddings with other types of molecular features is presented in Section 3.3.

### 3.3 Comparison of MACAW Embeddings and Conventional Molecular Descriptors

Conventional molecular descriptors can be considered a type of molecular embedding since they map molecules onto a numerical space, which may be continuous or discrete. Many conventional two-dimensional (2D) descriptors are obtained from algebraic operations over graph representations of molecules. While some of the most widely used tools to compute descriptors involve commercial packages, like alvaDesc, Dragon, or MOE, some free alternatives are available, such as rdkit, ChemDes,^6^ Mordred,^7^ or PaDEL.^41^ One challenge with conventional molecular descriptors is that a given descriptor may be useful for one problem but not for another. Besides, many descriptors suffer from collinearities, or the algorithms to compute them break when applied to different types of molecules. Thus, considerable feature cleaning and selection work is often needed before they can be used for modeling purposes. Furthermore, conventional molecular descriptors may define a rugged high-dimensional embedding, since some descriptors are discrete or may vary by orders of magnitude, so they may be challenging to use for inverse design tasks. Here, we compare MACAW embeddings with the rdkit and alvaDesc molecular descriptors, Chemical Checker (CC) signatures, and mol2vec embeddings.

**Figure 5:**
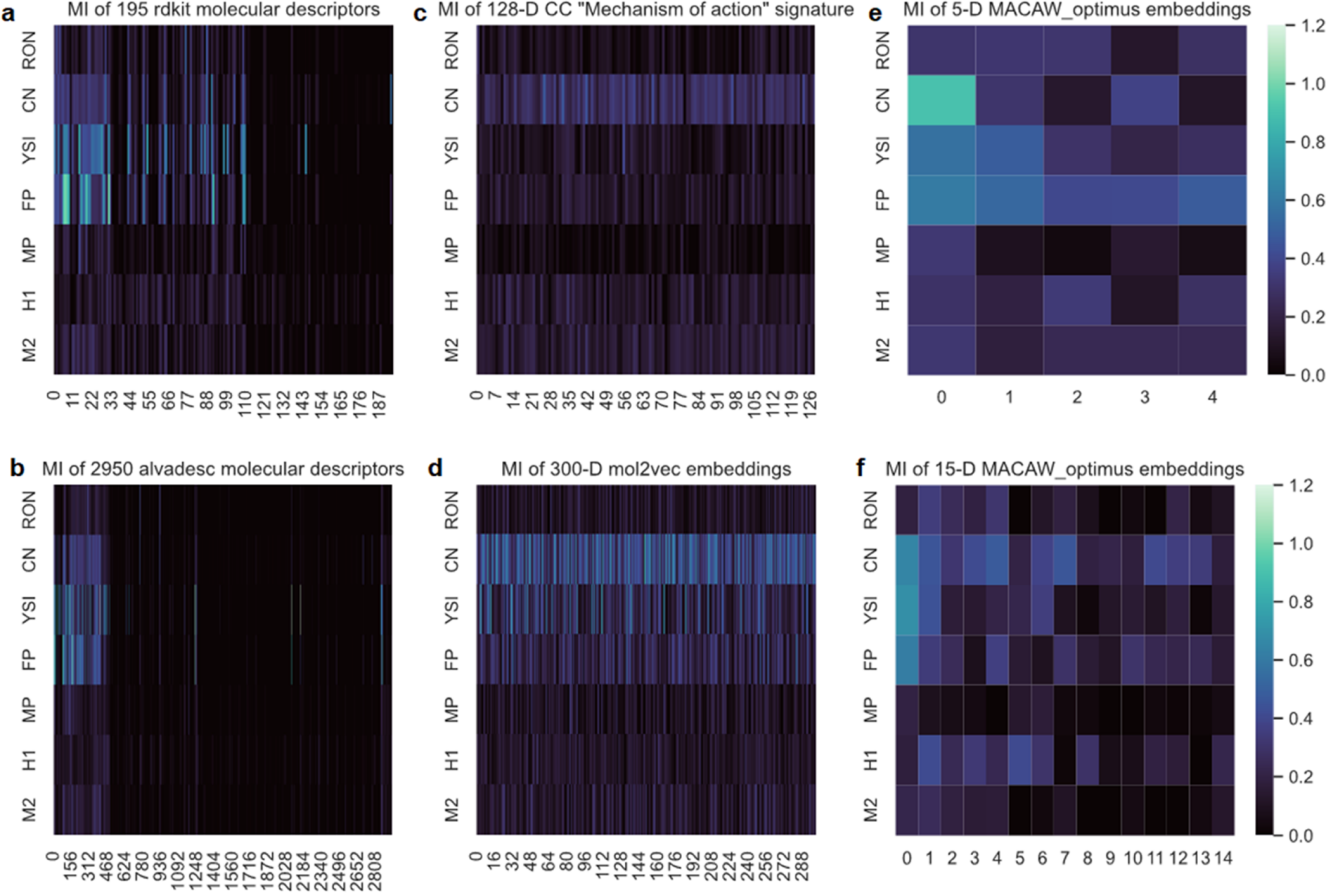
MACAW embeddings capture molecular information useful to describe a variety of properties more effectively than conventional molecular descriptors. The heat maps show the mutual information (MI) for regression between every feature (horizontal axis) and the target variable (vertical axis) for seven datasets in this work; (a) 195 molecular descriptors computed using rdkit 2020.09.4 after removing invalid descriptors; (b) 2950 molecular descriptors computed using alvaDesc 2.0.2 after removing invalid descriptors; (c) 180-D CC 3D fingerprint signatures; (d) 180-D CC MOA signatures; and (e) 5-D MACAW embeddings and (f) 15-D MACAW embeddings using the default MACAW_optimus settings. MACAW embeddings exhibit a relatively high mutual information in the different datasets compared to the conventional molecular descriptors, and all of the dimensions of the MACAW embedding tend to remain relatively informative.

The mutual information (MI) quantifies how informative a given descriptor is of a variable of interest (Figure 5). MI can be regarded as a generalization of the correlation coefficient. It takes values equal to or greater than zero, with higher values indicating a stronger association between the variables. Unlike the linear correlation coefficient, however, the mutual information captures information about all-dependence between two variables, both linear and nonlinear.^33,34^

A given conventional molecular descriptor (Figure 5a) can be informative for some problem datasets (high MI, clear color) but not useful in another (low MI, dark color). Thus, identifying those descriptors useful for a given task out of the many descriptors available (variable selection) can be a challenge. This process tends to be time-consuming, as it generally involves evaluating and comparing models trained with different subsets of the descriptors (see Jupyter Notebook 5). Some cheminformatic packages allow to compute more descriptors than others (Figure 5b), but this may not necessarily translate into better results and can further complicate the variable selection. Moreover, feature cleaning is often necessary when working with conventional molecular descriptors. Of note, out of 4179 molecular descriptors implemented in the software alvaDesc, a state-of-the-art commercial package, 1233 could not be calculated for some molecules in the dataset and had to be dropped. On the other hand, Figure 5 also suggests that modeling the molecular binding affinities to the histamine H1 or muscarinic M2 receptors may be more challenging than modeling the cetane number or flash points using these features, as their mutual information is in general lower.

A variety of alternative molecular representations have been proposed recently, providing pretrained molecular embedders. For example, mol2vec^42^ provides 300-D embeddings of molecules resulting from regarding molecules as sentences and substructures as words and deriving an embedding from a very large chemical library (19.9 M compounds) in an approach inspired by word2vec.^43^ As another notable example, Chemical Checker (CC) signatures have been proposed as a method to embed molecules in a continuous numerical space.^44,45^ Twenty-five different 128-D embeddings or signatures can be computed for each molecule, which differ in the amount and nature of the molecular property data that was used to train the specific embedder. Around 800,000 small molecules and associated data were used in the construction of the embedders. Results for two different CC embeddings are illustrated in Figure 5c,d. Unlike MACAW embeddings, the dimensionality of CC signatures and mol2vec embeddings is predetermined, and the embedders are pretrained. Their relatively high dimensionality suggests that additional feature selection may be beneficial for small datasets, as some dimensions may be irrelevant for the modeling problem at hand (Figure 5).

MACAW embeddings (Figure 5e) seem to capture relevant molecular information more consistently across the different datasets than conventional molecular descriptors, which is expected to facilitate predictive modeling (see below). This is indicated by the MI values being comparatively high and uniform for a given problem dataset. Note that MACAW’s performance can be further optimized to each problem by fine-tuning its hyperparameters, such as the number of landmarks or the type of fingerprint and molecular similarity metric used. On the other hand, when increasing the dimensionality of the MACAW embeddings 3-fold (Figure 5f), useful chemical information is spread across the different dimensions, so that they all tend to remain informative. This is illustrated by the MI values remaining comparatively high even after increasing the dimensionality of the embedding. Thus, MACAW embeddings avoid the feature selection step that is often needed for conventional molecular descriptors, expediting the modeling process.

MACAW embeddings allow us to train models that perform similarly or better than models trained on conventional molecular descriptors without the need for feature selection, saving significant time (Figures 4 and S4). A subset of conventional molecular descriptors was selected to model each molecular property through a variable selection algorithm (see Section 2). Different descriptors were selected for different datasets, in line with the observation that a given descriptor tends to not perform well across different properties. We managed to train SVM models on the selected descriptors that offered very reasonable predictive performances (Figure S4 and Jupyter Notebook 5). Notwithstanding, the predictive performances of models trained on conventional descriptors were matched or improved upon by similar models trained on MACAW embeddings (Figures 4 and S4). This agrees with the observation that MACAW embeddings tend to be more informative than most conventional molecular descriptors (Figure 5).

Another positive aspect of MACAW is that the embedding is defined by the input data, and thus it is not limited to a pretrained representation, enabling the flexible modeling of diverse properties and specific regions of the chemical space. We explored the use of different 128-D CC signatures as replacements for conventional descriptors, including a 15-D variable selection step. We noticed that the choice of the signaturizer can have a significant effect on the performance of the resulting predictor, and choosing the optimal one is not trivial (Figure S5 and Jupyter Notebook 6). Similarly, we explored the use of 300-D mol2vec embeddings as a replacement for conventional descriptors with a 15-D variable selection step (Figure S6). When compared to the performances offered by MACAW embeddings, we find that the lower dimensionality of MACAW embeddings and their definition based on the chemical subspace relevant to the task at hand are well-suited to the small-size datasets illustrated in this work, which are commonly found in the biosciences.

### 3.4 Virtual Screening for Molecules with Desirable Properties

**Figure 6:**
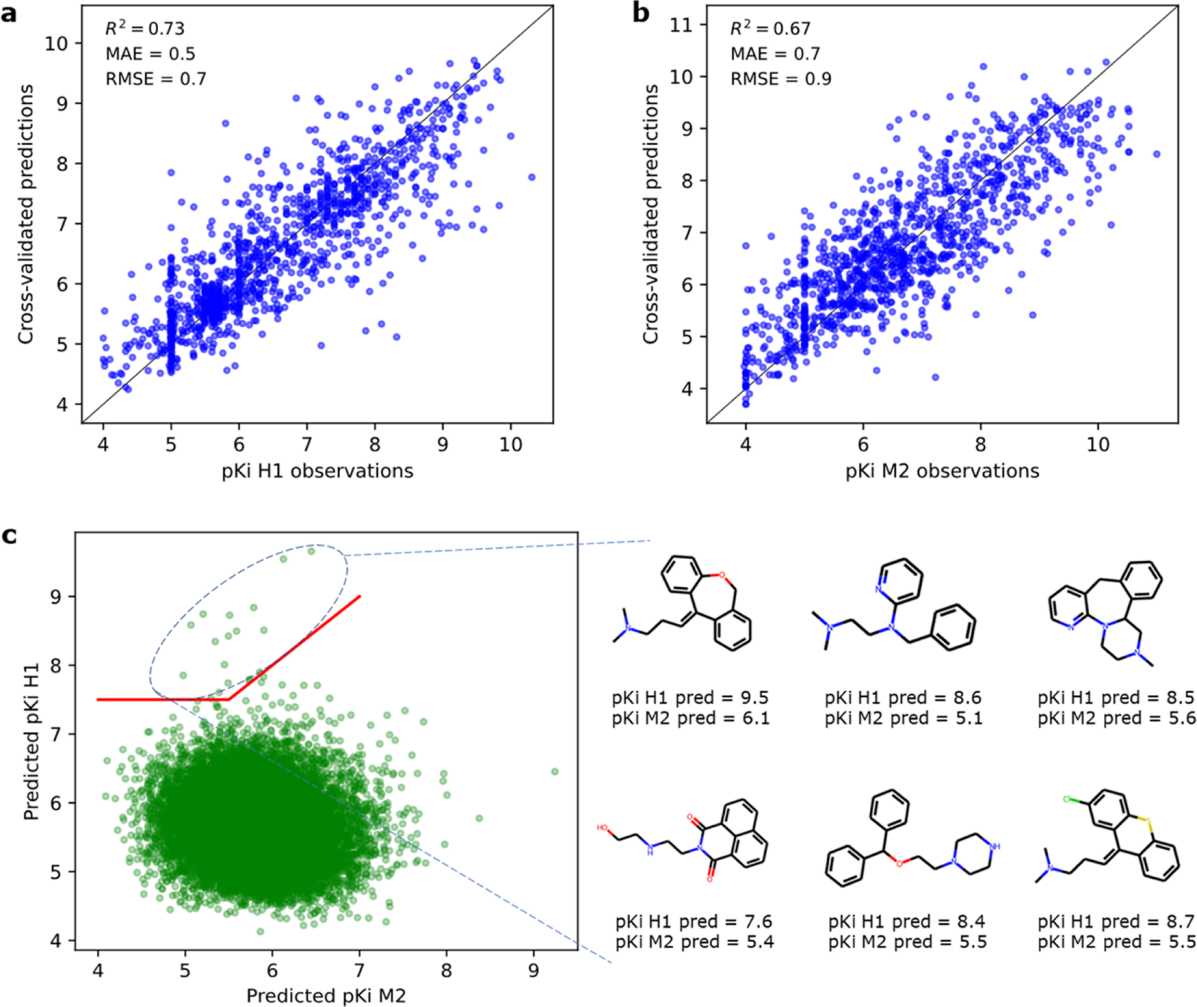
MACAW embeddings may help identify molecules with high binding affinity to the histamine H1 receptor and limited affinity to the muscarinic M2 receptor. (a) Parity plot of the H1 receptor binding model. (b) Parity plot of the M2 receptor binding model. (c) Virtual screening of a custom library (19,490 molecules) defining the region of interest. Some promising molecules with high predicted binding affinity and specificity for the histamine H1 receptor are also indicated. See Jupyter Notebook 3 for details.

MACAW can be applied to rapidly embed large molecular libraries, enabling virtual screening (Figure 6). In virtual screening, large, predefined catalogs of molecules are evaluated computationally to facilitate the discovery of chemical matter suitable for a given application. To illustrate this, we trained models using MACAW embeddings to predict the binding affinity of compounds to the histamine H1 receptor, a well-known pharmaceutical target^46,47^ (Figure 6a). A separate model was similarly trained to predict binding affinity to the muscarinic M2 receptor, a related protein considered a potential off-target. High binding affinity to the H1 receptor is desired, as it is a prerequisite for pharmacological activity (target inhibition), whereas low binding affinity to the M2 receptor is desired, as the compound may otherwise lead to undesired side effects.

Both binding affinity models were trained on newly generated experimental data for this work, which was embedded using MACAW. Afterward, the models were exhaustively applied to a custom virtual library of molecules to predict binding affinities to both receptors. Promising virtual hits could be identified, which showed a high predicted binding affinity to the H1 receptor and considerably lower predicted binding affinity to the M2 receptor (Figure 6b). Some of these virtual hits are illustrated in Figure 6c and represent excellent starting material for experimental tests.

In cases where the predictive model is expensive to evaluate (e.g., a kernel-based model trained on a large dataset), the functions hit_finder and hit_finder_grad in the MACAW package allow searching for promising molecules across an embedded library without having to exhaustively evaluate all of the molecules. hit_finder_grad uses a multistart gradient-based minimization algorithm, which is suitable for smooth models, as it is often the case for those trained on MACAW embeddings. By contrast, hit_finder does not estimate the gradient of the predictive model function; it only assumes that molecules with similar property values lie in similar regions of the embedding space. A diagram of the hit_finder algorithm is shown in Figure S2. Used examples of these functions are provided in Jupyter Notebook 4.

### 3.5 Inverse Molecular Design

MACAW embeddings can also help with the generation of molecules de novo satisfying a given property specification (inverse molecular design). Several approaches can be envisioned for this purpose. For example, since embedding arbitrary input molecules using MACAW is quite fast, it may be possible to generate large molecular libraries, embed them, and train a neural network to act as a decoder.^8^ Since the embedding is not being trained, this process might be even done in an active learning fashion. However, one advantage of MACAW is that it avoids the complexity of training an encoder network. Thus, here we report an approximate strategy that does not require training a decoder either and that directly provides molecules matching a desired property specification. The approach is summarized in Figure 2 and is discussed next.

A prerequisite for inverse molecular design is the ability to generate new molecules, and there is much interest in generating new molecules based on small datasets.^9,11^ In MACAW, molecular generation from small datasets is achieved efficiently using the library_maker function. The generative algorithm leverages the robustness of the SELFIES molecular representation,^12^ which allows concatenating random combinations of a set of SELFIES symbols and decoding them into SMILES strings with ∼100% validity. In MACAW’s library_maker, the choice of symbols is not totally random, but it is informed by the set of input molecules provided so that the molecules generated are “centered” to some extent around the input distribution of molecules. In particular, molecules are generated in a probabilistic manner based on the distribution of one-hot SELFIES representations observed in the input molecules after adding some stochasticity. By default, the transition probabilities observed between two SELFIES tokens are used in the generative process but other options are available (see Section 2). The library_maker function also filters the resulting library to avoid duplicates and synonyms. The output is a list of molecules in canonical SMILES format. Libraries of 10^4^–10^6^ molecules can be generated in seconds to minutes in a laptop computer (4 cores, 4 GB RAM) using this approach (Figure S7).

Besides generating new molecules, it would be desirable to be able to control the focus or spread of the molecular distribution generated.^48^ MACAW allows a facile control of this focus by setting the noise_factor argument with values between 0 and 1. An illustration of the effect of this parameter on the molecules generated is shown in Figure S7 as a uniform manifold approximation and projection (UMAP).^49^ Notably, MACAW’s algorithm allows generating distributions of molecules around input datasets as small as 10^2^ molecules with adjustable focus.

A computational directed evolution approach to inverse molecular design is proposed, which builds on top of MACAW’s molecular generator (Figure 2). The approach is implemented in MACAW’s library_evolver function. It requires a MACAW embedder and a predictive property model, as well as the ability to generate new molecules centered around a given set of molecules (provided by the library_maker tool described above). First, *k*1 molecules (3000 by default) are generated throughout the embedding space based on the input dataset. Then, the molecules are embedded using MACAW, their property values are predicted, and the *k*2 (100 by default) most promising ones (i.e., those with predicted property values closest to the desired specification) are identified. These *k*2 molecules are then used to inform a new molecular generation round. The *k*2 most promising molecules from one round are also carried over to the next round. The process is repeated several rounds (*n*_rounds = 8 by default), and the most promising molecules in the final round (*n*_hits = 10 molecules by default) are returned to the user in SMILES format, along with their predicted property values. The accompanying Jupyter Notebook 4 illustrates the use of the library_evolver function, among others.

MACAW is able to generate compounds that fit the desired specification following the molecular generation strategy introduced. For illustrative purposes, we requested the design of molecules with three different RON specifications: 40, 80, and 120. The tool succeeds at proposing diverse molecules whose predicted properties satisfy the desired design specification (Table 1). Although some chemical properties beyond what the training dataset can teach may not be properly captured, the outputs are by and large very consistent with domain-specific knowledge:^24,25,50^ molecules that are longer, with lower branching, and fewer unsaturations are proposed to achieve a low RON specification (i.e., a low antiknocking capacity), whereas shorter molecules, branched, with unsaturations and/or oxygenated are proposed when requesting a high RON specification.

**Table 1:**
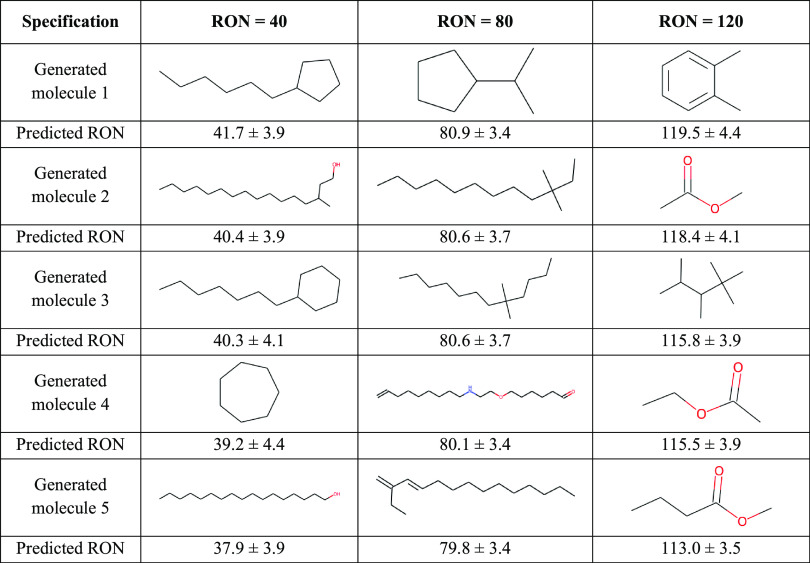
MACAW’s Library_evolver Tool Enables the Directed Evolution of Molecules with Prespecified Properties In Silico, Like the Research Octane Number (RON)^a^

a A limited set of molecules with known RON was used to train a relevance vector regressor (RVR), a type of machine-learning model (Jupyter Notebook 4). The tool generates a library of molecules around the input molecules, selects a subset close to the desired specification, and then uses the updated subset to generate a new library more focused on the promising regions of the chemical space. The table illustrates the outputs after eight library generation iterations for different RON specification values. The errors represent the standard deviation of predictive distribution learned by the RVR at the query points. See Jupyter Notebook 4 for details.

MACAW can also address multiple design specifications simultaneously by combining them in a single objective function and providing it as the input model to the library evolver. Jupyter Notebook 4 illustrates one way to consider the prediction uncertainty and the synthetic accessibility^51^ in the recommendation of new molecules. Future research will explore additional features for MACAW updates, such as the incorporation of other similarity metrics and kernels^52^ for the computation of molecular distances, new projection methods, the use of parallelization, or a model-based decoder as an alternative for the inverse design of new molecules.

## 4 Conclusions

In this work, we propose MACAW, a novel algorithm for molecule embedding and generating molecules that meet a desired property specification. The MACAW low-dimensional embeddings are rich in structural information, fast to compute, and tuned to the molecular dataset at hand. MACAW embeddings are obtained through a fast multidimensional scaling approach focused on a few landmark molecules, followed by projection of the remaining (nonlandmark molecules) onto the embedding space via triangulation (Figure 1). The use of landmark embedding methods combined with an improved landmark selection strategy allows for a high-quality embedding at a low computational cost. Notably, instead of computing *N × N* distances between the *N* query molecules, we only need to compute *N × L* molecular distances between the queries and the landmarks.

The embeddings can be used as a replacement for conventional descriptors in modeling molecular properties and virtual screening, without the need for variable cleaning and selection. MACAW embeddings are shown to perform favorably compared to conventional molecular descriptors, simplifying the modeling, saving time, and improving the accuracy of the models trained on them. The speed of MACAW also allows its application to large molecular libraries for use in virtual screening applications.

Besides enabling the prediction of molecular properties, MACAW can solve inverse design problems. For this, we created a molecule generator algorithm based on SELFIES that is fast and efficient. The molecule generator performs well even in very small datasets and allows certain control on the focus of the molecular distribution being generated. MACAW generates molecules in a probabilistic manner from a given set of molecules, considering either the probability of symbols as a function of their absolute position in the molecular string or the probability of transitions between consecutive symbols. The resulting molecule generator can be coupled with a selection step based on a molecular property of interest. This allows it to automatically evolve focused molecular libraries toward the desired property specification. Thus, we believe that MACAW will be a useful addition to the cheminformatic toolkit for molecular modeling and inverse design in synthetic biology, chemistry, and engineering. It also represents a welcome addition to existing retrobiosynthesis tools by not only helping predict properties but also suggesting molecules that exhibit the desired property.

## Supporting Information Available

MACAW’s library_maker algorithm allows generating libraries of molecules given an input list of molecules (Figure S1); illustration of MACAW’s hit_finder algorithm, which allows identifying promising hits from a library without having to exhaustively evaluate the predictive model on all the molecules (Figure S2); effect of MACAW hyperparameters on the performance of SVR models trained on the resulting MACAW embeddings (Figure S3); predictive performance of conventional rdkit molecular descriptors in the different datasets in this work (Figure S4); predictive performance of features extracted from six different Chemical Checker signatures in the RON dataset (Figure S5); predictive performance of features extracted from mol2vec embeddings trained on 19.9 M compounds (Figure S6); MACAW’s library_maker tool allows the rapid generation of molecular libraries with variable diversity from small molecular datasets (Figure S7) (PDF)

## Notes

The different datasets used in this work are provided as csv files in the link: https://github.com/LBLQMM/MACAW/tree/main/notebooks/data

## Notes

A Python implementation of MACAW is available in the repository: https://github.com/LBLQMM/MACAW/ Examples of use are provided as well as Jupyter notebooks. MACAW has been developed under Python 3.8.8 and it relies on packages: numpy 1.19.4, scikit-learn 0.24.1, rdkit 2020.03.6, scipy 1.6.2, and selfies 2.0.0. The accompanying Jupyter Notebook 1 demonstrates the use of the MACAW library to describe molecules and train regression models of different molecular properties, Jupyter Notebook 2 illustrates the modeling of biofuel properties, Jupyter Notebook 3 contains the modeling and virtual screening against the H1 and M2 receptors, Jupyter Notebook 4 contains the de novo design of molecules based on RON specifications, and Jupyter Notebooks 5, 6, and 7 illustrate some models using conventional molecular descriptors, CC signatures, and mol2vec embeddings, respectively. These notebooks can be accessed at https://github.com/LBLQMM/MACAW/tree/main/notebooks.
