# Supplementary material for: MACAW: An Accessible Tool for Molecular Embedding and Inverse Molecular Design
Source: J Chem Inf Model. 2022 Jul 20;62(15):3551–64. doi: 10.1021/acs.jcim.2c00229 (PMC9364320; doi:10.1021/acs.jcim.2c00229)
Supplement: Supplementary file 1 — ci2c00229_si_001.pdf [file ci2c00229_si_001.pdf]

SUPPORTING INFORMATION

MACAW: an accessible tool for molecular embedding and inverse molecular design

Vincent Blay[a,b], Tijana Radivojevic[a,b,c], Jonathan E. Allen[d], Corey M. Hudson[e], Hector Garcia Martin[a,b,c,*]

[a] Biological Systems and Engineering Division, Lawrence Berkeley National Laboratory, Berkeley, CA, 94720, USA.
[b] Biofuels and Bioproducts Division, DOE Joint BioEnergy Institute, Emeryville, CA, 94608, USA.
[c] DOE Agile BioFoundry, Emeryville, CA, 94608, USA.
[d] Global Security Computing Applications, Lawrence Livermore National Laboratory, Livermore, 94550 California, USA.
[e] Sandia National Laboratories, Livermore, 94550 California, USA.

* hgmartin@lbl.gov

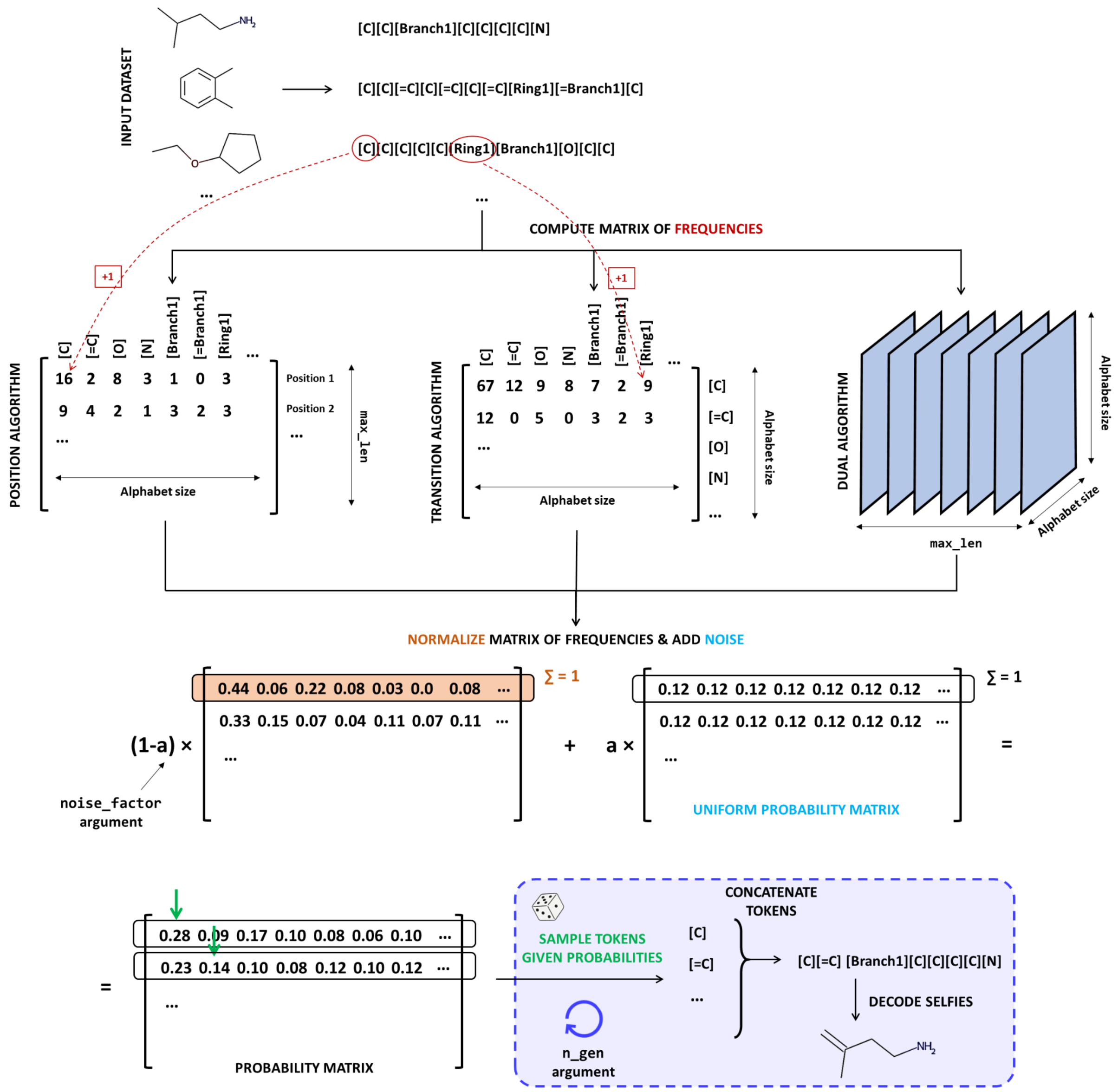

Fig. S1. MACAW's `library_maker` algorithm allows generating libraries of molecules given an input list of molecules. The input molecules are encoded as SELFIES and they are used to populate a matrix of frequencies or counts. By default, only the SELFIES tokens observed in the input list are considered to make up the SELFIES alphabet. Depending on the algorithm chosen, the frequency matrix will capture i) counts of tokens as a function of their position in the SELFIES string (default `position` algorithm), ii) counts of tokens following each specific token (`transition` algorithm), or iii) counts of tokens in a specific position of the SELFIES word after another specific token (`dual` algorithm, in which case the matrix is 3-dimensional). In the `position` and `dual` algorithms, the frequencies of the first token in each word are considered separately, since they are not preceded by any other token. After computing the matrix of frequencies, the matrix is normalized row-wise and noise is added, resulting in a matrix of probabilities. The user can tune the level of noise with the argument `noise_factor`. The probability matrix can be used to generate as many molecules as desired (dashed purple box), specified with the argument `n_gen`. For this, tokens are sampled based on the probabilities in the matrix. In the `position` and `dual` algorithms, tokens are sampled one at a time for each molecule, since having drawn a token affects the probability of drawing the next token. The number of tokens being sampled to make each molecule is drawn from a discrete distribution up to the specified `max_len` (defaults to the length of the longest SELFIES in the input). Tokens are concatenated and the resulting SELFIES string is decoded as a valid molecule.

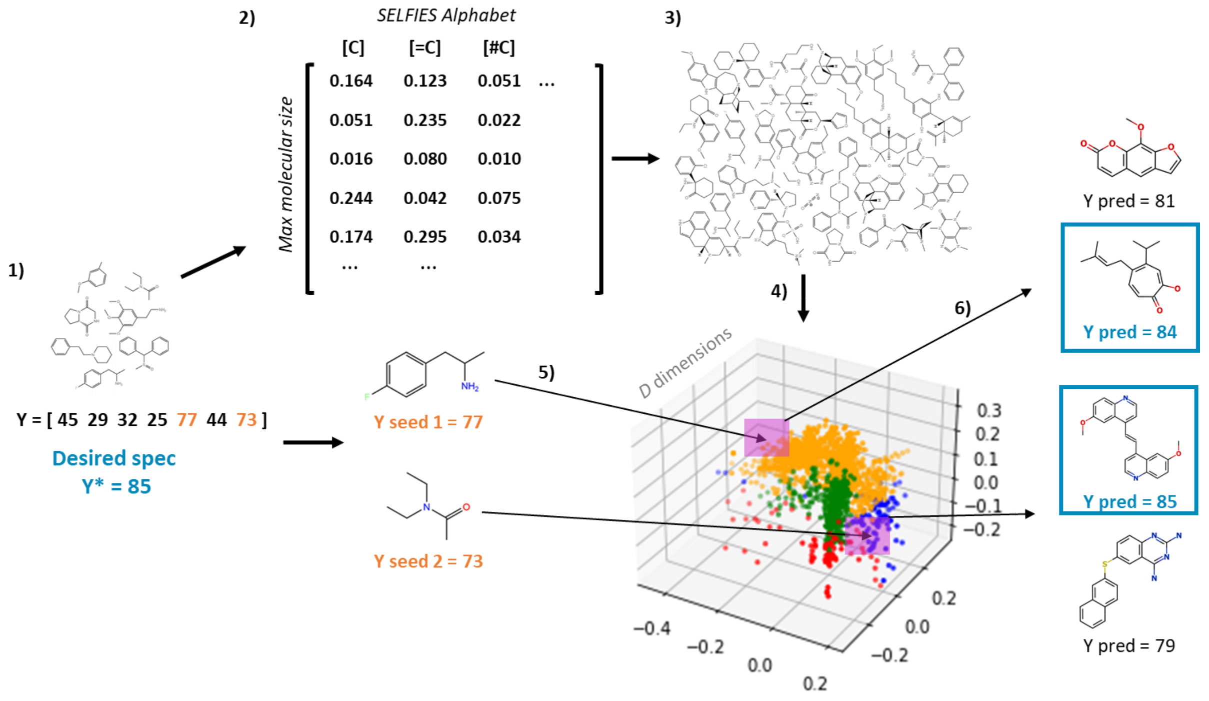

Fig. S2. Illustration of MACAW's `hit_finder` algorithm, which allows identifying promising hits from a library without having to exhaustively evaluate the predictive model on all the molecules. It requires a dataset relevant to the problem, the corresponding property values, and the desired design specification (1), as well as the MACAW embedder and predictive property model. The input molecules are used to generate a noisy probability matrix (2), from which large numbers of molecules can be generated (3). The library molecules are projected using MACAW onto a *D*-dimensional embedding space. The embedded library is parceled using a BallTree strategy for fast search (note colored points) (4). The k_1 most promising input molecules (5) are used to retrieve k_2 promising candidate molecules from the vicinity of each one in the MACAW space (note purple boxes) (6). The candidate molecules are evaluated using the property predictive model and the most promising molecules are returned based on the specification (blue boxes). In this illustration, $k_1 = 2$ and $k_2 = 2$.

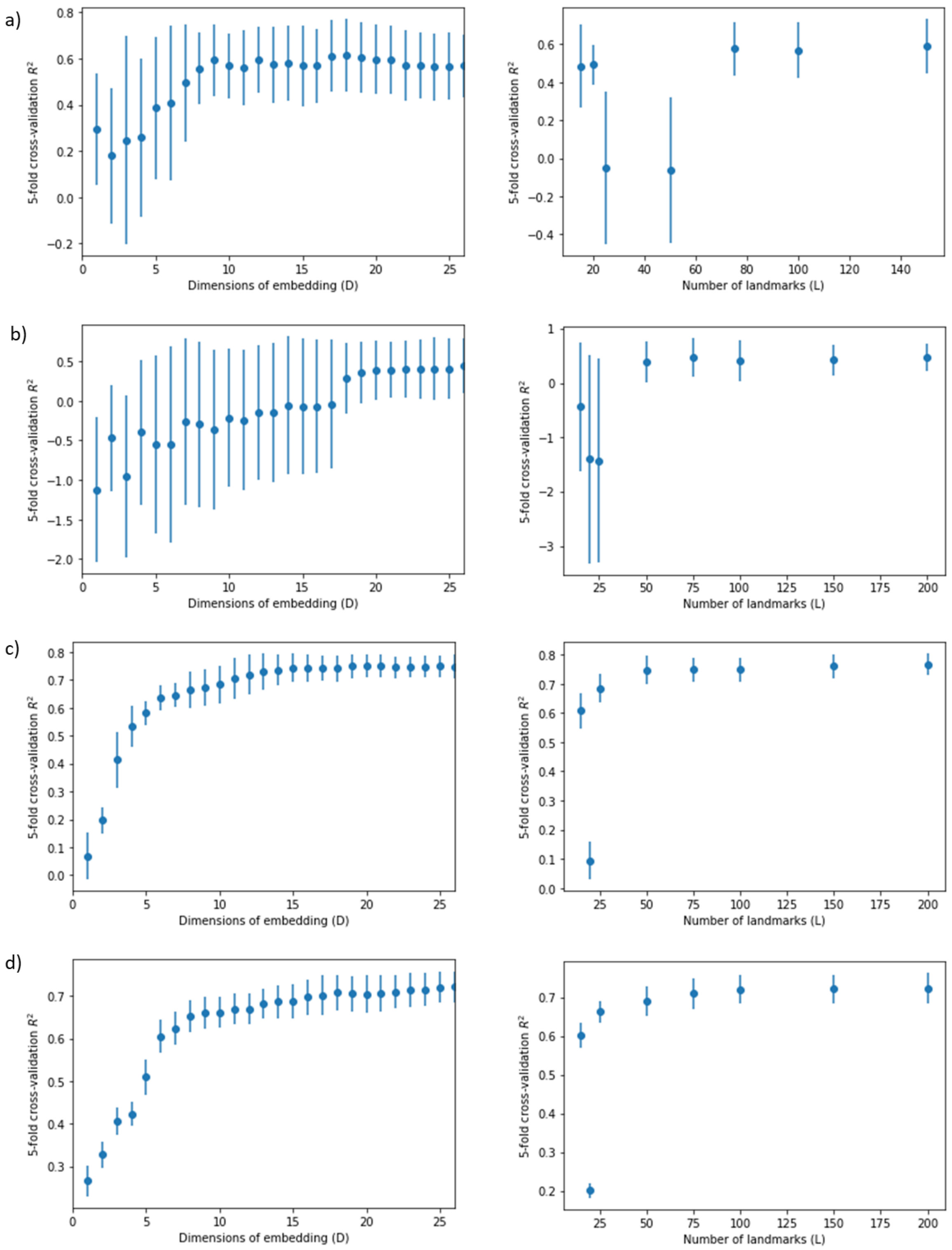

Fig. S3. Effect of MACAW hyperparameters on the performance of SVR models trained on the resulting MACAW embeddings. Models of different properties are shown: a) RON, b) flash point, c) histamine H1 receptor binding affinity, d) muscarinic M2 receptor binding affinity. In all cases, the MACAW embeddings were computed using "atompairs" as the fingerprint type and "tanimoto" as the distance metric. The entire datasets were used and performance of the model was assessed by looking at the R^2 scores in 5-fold cross-validation.

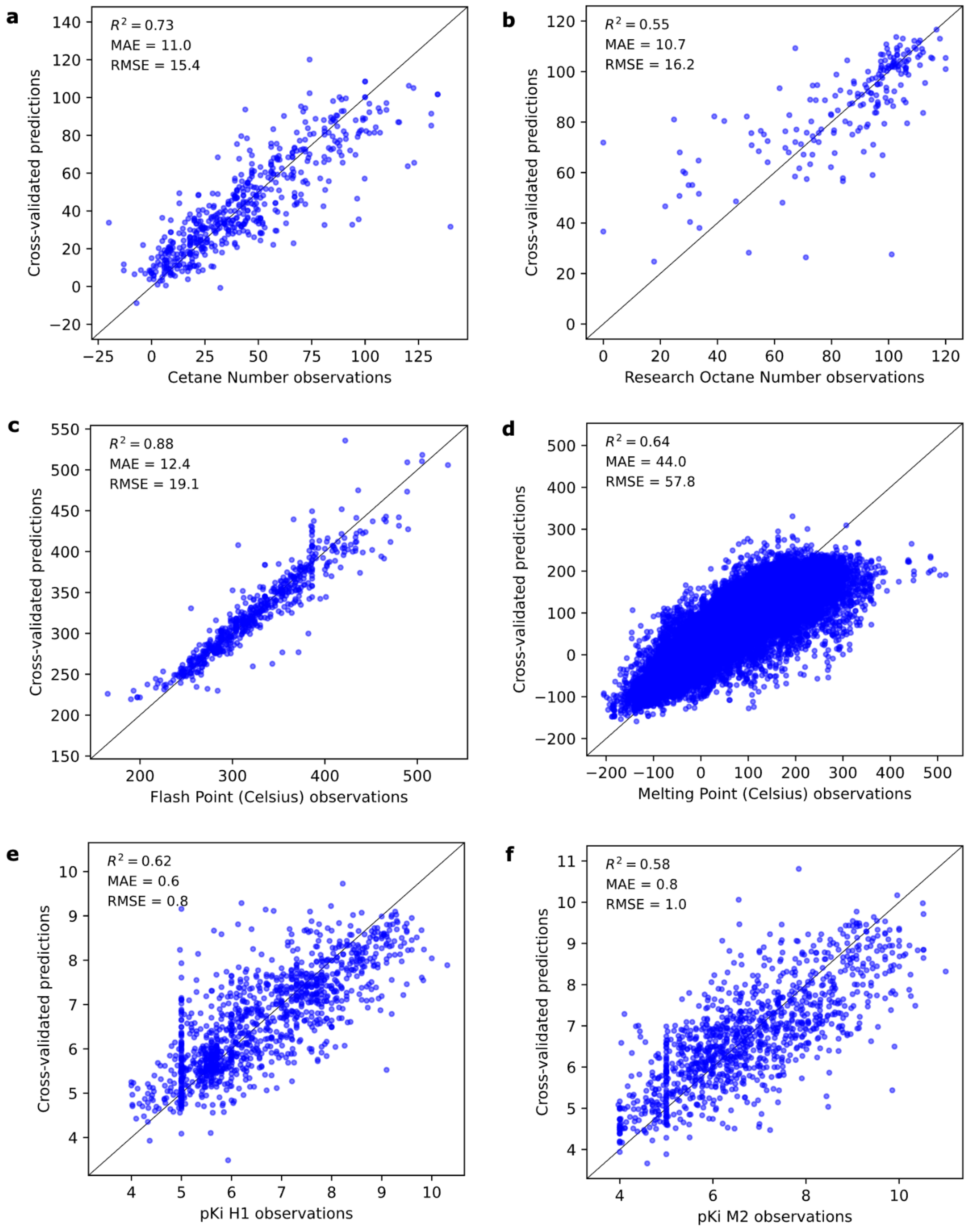

Fig. S4. Predictive performance of conventional rdkit molecular descriptors in the different datasets in this work. The top 15 conventional molecular descriptors were selected for each problem using sklearn's `SequentialFeatureSelector` algorithm using multiple linear regression during the selection. The features selected were then used to train support vector regressors, whose results are shown in the parity plots (Jupyter Notebooks 2 and 3). See Jupyter Notebook 5 for details. Compare with prediction in Fig. 3 enabled by the MACAW embedding.

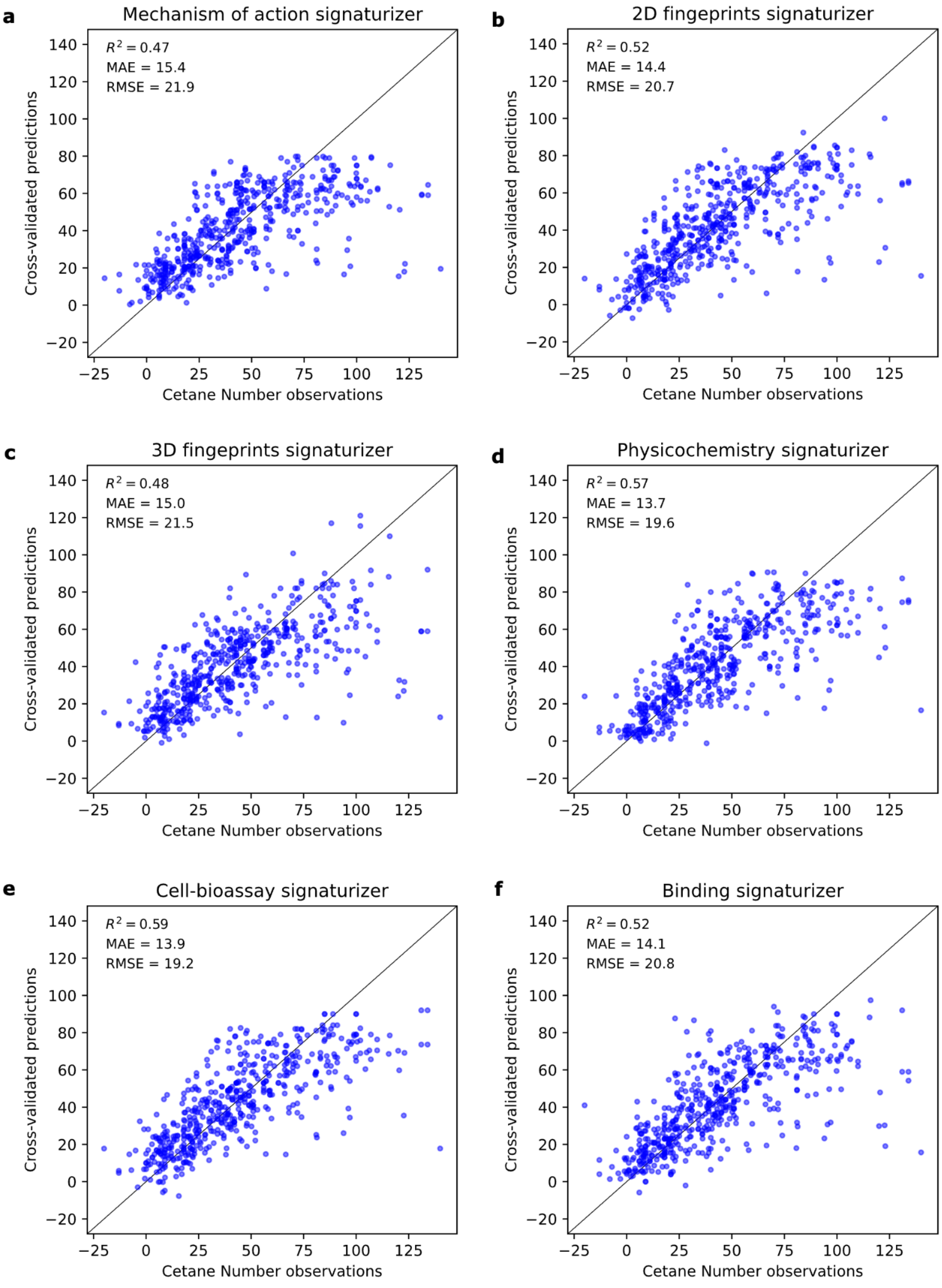

Fig. S5. Predictive performance of features extracted from 6 different Chemical Checker signatures in the RON dataset. The 15 most informative dimensions of each 128-D signature were extracted using sklearn's `SequentialFeatureSelector` algorithm using multiple linear regression during the selection. The features selected were then used to train support vector regressors, whose results are shown in the parity plots. See Jupyter Notebook 6 for details.

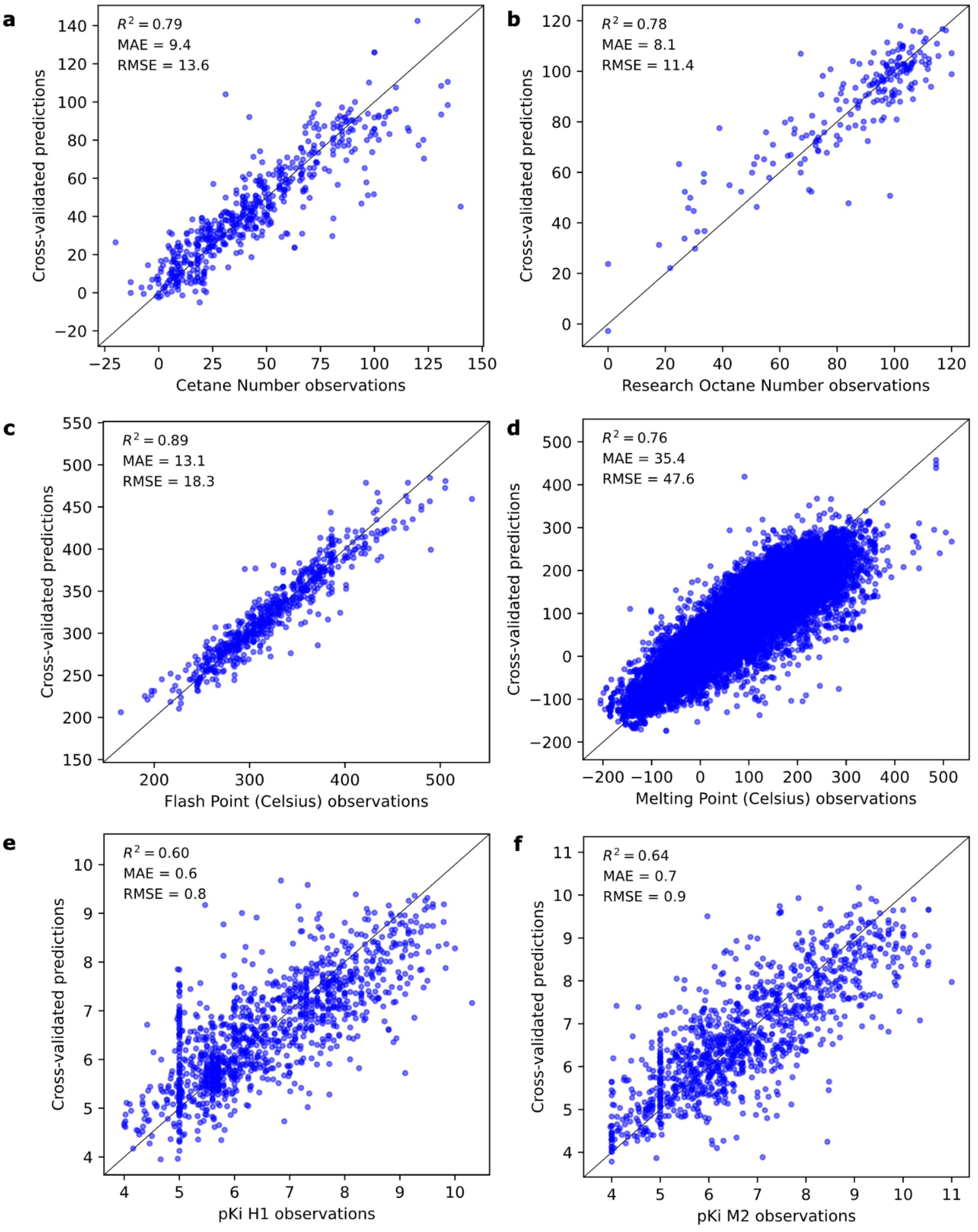

Fig. S6. Predictive performance of features extracted from mol2vec embeddings trained on 19.9 M compounds. The 15 most informative dimensions of each 300-D embedding were extracted using sklearn's `SequentialFeatureSelector` algorithm using multiple linear regression during the selection. The features selected were then used to train support vector regressors, whose results are shown in the parity plots for 6 different datasets in this work. See Jupyter Notebook 7 for details.

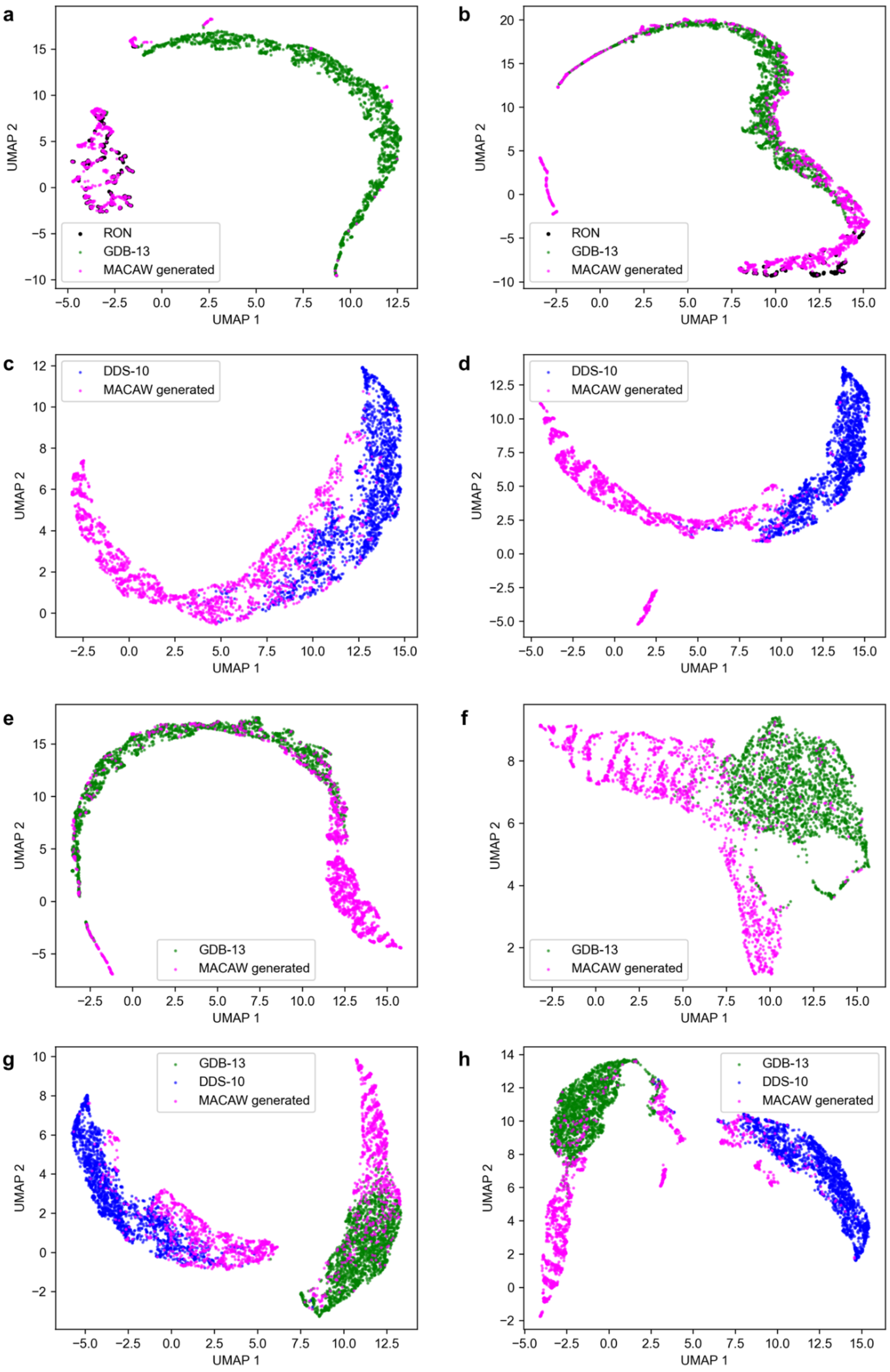

Fig. S7. MACAW's `library_maker` tool allows the rapid generation of molecular libraries with variable diversity from small molecular datasets. In a) we primed the algorithm with the small RON dataset, whereas in b) we primed the algorithm with both the RON dataset and a sample from the GDB-13 database (https://gdb.unibe.ch/downloads/). The same algorithm settings were used to produce a) and b) (noise_factor=1e-5, p="emp"). In c) and d) we used the same dataset to prime `library_maker`, a sample from Enamine's Diversity Set 10 (DDS-10, https://enamine.net/compound-libraries/diversity-libraries). In c) we set noise_factor=1e-4, whereas in d) we set noise_factor=0.3. In e) and f) we used the same noise_factor=0.3 but set the algorithm to "position" in e) and to "transition" in f). In g) and h) we combined two diverse samples from GDB-13 and DDS-10 to prime `library_maker`. In g) noise_factor=1e-4 was used, while h) shows the results for noise_factor=0.3. The settings p="exp" and algorithm="position" were used by default unless indicated otherwise. All points in each plot were used to compute the corresponding 2D UMAP projection using umap-learn 0.5.1 in Python. For this, ca. 200 rdkit descriptors were used to featurize the molecules. The results illustrate the ability of MACAW's `library_maker` tool to generate diversity across a broad chemical space but also to focus it on a much smaller chemical subspace, depending on the input dataset and the settings used.
